# Transcriptomics and metabolomics reveal tolerance new mechanism of rice roots to Al stress

**DOI:** 10.3389/fgene.2022.1063984

**Published:** 2023-01-10

**Authors:** Jingbo Wang, Chang Su, Zhibo Cui, Lixiang Huang, Shuang Gu, Sixu Jiang, Jing Feng, Hai Xu, Wenzhong Zhang, Linlin Jiang, Minghui Zhao

**Affiliations:** Rice Research Institute, Collaborative Innovation Center for Genetic Improvement and High Quality and Efficiency Production of Northeast Japonica Rice in China, Shenyang Agricultural University, Shenyang, China

**Keywords:** aluminum tolerance, rice, internal tolerance, transcriptomics, metabolomics

## Abstract

The prevalence of soluble aluminum (Al) ions is one of the major limitations to crop production worldwide on acid soils. Therefore, understanding the Al tolerance mechanism of rice and applying Al tolerance functional genes in sensitive plants can significantly improve Al stress resistance. In this study, transcriptomics and metabolomics analyses were performed to reveal the mechanism of Al tolerance differences between two rice landraces (Al-tolerant genotype Shibanzhan (KR) and Al-sensitive genotype Hekedanuo (MR) with different Al tolerance. The results showed that DEG related to phenylpropanoid biosynthesis was highly enriched in KR and MR after Al stress, indicating that phenylpropanoid biosynthesis may be closely related to Al tolerance. E1.11.1.7 (peroxidase) was the most significant enzyme of phenylpropanoid biosynthesis in KR and MR under Al stress and is regulated by multiple genes. We further identified that two candidate genes* Os02g0770800* and *Os06g0521900* may be involved in the regulation of Al tolerance in rice. Our results not only reveal the resistance mechanism of rice to Al stress to some extent, but also provide a useful reference for the molecular mechanism of different effects of Al poisoning on plants.

## Background

The prevalence of soluble aluminum (Al) ions is one of the major limitations to crop production worldwide on acid soils (von Uexkull and Mutert, 1995). Al isn’t toxic to plants in soils with a normal pH, because it is usually stabilized by insoluble compounds, such as silicates, sulfides, and silicon dioxide. However, in acidic soils with pH ≤ 5.5, Al ions enter the root system as free Al ions and are toxic to plants, of which trivalent Al^3+^ is the most toxic (Kochian et al., 2004; Hiradate et al., 2007; Chauhan et al., 2021). Rice (*Oryza sativa*) is among the most important food crops in the world and provides food to nearly half of the world’s population. In acidic soil, micromolar Al^3+^ can exert toxic effects on rice roots within a short period, which limits plant growth and the absorption of nutrient elements, and results in a decrease in grain yield (Ma et al., 2002; Huang et al., 2009; Wang et al., 2021). In recent years, the problem of soil acidification has also increased (Goulding, 2016), which seriously affects the stability of rice yields (Gracas et al., 2021). Among the most effective solutions to increase tolerance to Al toxicity are to screen and utilize Al-tolerant germplasm resources, and to finely map and clone Al-associated genes. In-depth exploration of the response and regulatory mechanism of rice to Al stress is a prerequisite and foundation for the molecular breeding of Al-tolerant rice.

The response mechanisms of plants to Al can be divided into two categories: external exclusion and internal tolerance (Kochian, 1995; Kochian et al., 2004; Fang et al., 2021). The external exclusion mechanism occurs when plants utilize the cell wall and other organs as a barrier to exclude Al^3+^ from the plant cell machinery and to avoid the phytotoxicity of Al ions. These detoxification methods mainly include organic acid chelation, cell wall fixation, and root barriers. The internal tolerance mechanism operates to change active Al entering the cell into an insoluble or slightly soluble form that exerts no harmful effect on plants through chemical and biological reactions; these mechanisms reduce the content of trivalent Al ions in the cells. This detoxification method is mainly dependent on the formation of stable complexes by complexing Al ions with organic compounds (mainly organic acids) in the cytoplasm (Koyama et al., 1999; Ishikawa et al., 2000; Ma, 2000; Ma et al., 2014). Organic acid anions, such as malate, citrate, and oxalate, play a role in the response of plants to Al stress (Ma et al., 2001; Kochian et al., 2015; Yang et al., 2008). In addition, both *OsFRDL2* and *OsFRDL4* play an important role in promoting rice Al tolerance by regulating citrate transport (Yokosho et al., 2016).

Plant root tips are considered to be the critical location of Al toxicity (Bennet, 1991; Clemens et al., 2013) and are highly sensitive to Al ions. The detoxification and tolerance of plants to Al toxicity are strongly associated with the cell wall. The cell wall is the first self-protection barrier in plant cells, and 30%–90% of the Al in the root system accumulates in the cell wall, which demonstrates that the cell wall plays a vital role in resistance to Al toxicity (Shweta et al., 2017). The cell wall consists of three major components cellulose, hemicellulose, and pectin that in combination with water and proteins form the extracellular matrix (Somerville, 2006; Rose and Lee, 2010; Scheller and Ulvskov, 2010). The pectin in the cell wall comprises a group of acidic heteropolysaccharides that mainly contain D-galacturonic acid and α-1,4-glycosides as well as l-rhamnose, D-galactose, and 12 types of monosaccharides. Pectin is the most important binding site of Al ions and galactose metabolism is closely associated with expansion of the cell wall during plant growth (Dopico et al., 1989; Konno and Tsumuki, 1993). Lignin, cellulose, and hemicellulose are the main components of the plant skeleton. The lignin content and structure in plants are affected by biotic and abiotic stresses (Moura et al., 2010). As an important component of tissue growth, lignin promotes the transport of water and minerals, and the secondary cell wall is significantly thickened under heavy metal stress; thus, the heavy metal content may be associated with the permeability of the cell wall (Moura et al., 2010). The synthesis of lignins and coumarins is dependent on the phenylpropanoid metabolic pathway (Fraser and Chapple 2011). Phenylalanine ammonia-lyase (PAL) and 4-coumarate-CoA ligase (4CL), as the crucial enzymes of phenylpropanoid metabolism, are the main enzymes involved in lignin-specific biosynthesis (Lacombe et al., 1997; Chen et al., 2020).

Evidence from genetic studies suggests that Al tolerance in plants is a quantitative trait controlled by more than one major gene and several minor genes (Nguyen et al., 2001; Ninamango-Cárdenas et al., 2003). Malate transporters (ALMTs) are associated with Al tolerance in plants. The *ALMT1* gene, which was the first ALMT family gene discovered in wheat and Arabidopsis (*Arabidopsis thaliana*), encodes an Al-activated transporter that induces malate secretion in the roots and Al tolerance is improved by decreasing the Al ion concentration in cells (Sasaki et al., 2004; Vives-Peris et al., 2020; Zhu et al., 2022). The Al^3+^-enhanced malate transporter homolog *AtALMT1* was subsequently identified in Arabidopsis (Kobayashi et al., 2007). Additional Al-tolerance genes, which are members of the Multidrug and Toxic Compound Extrusion (MATE) transporter family, have been identified in rice, Arabidopsis, wheat, barley, rye, maize, and sorghum. *MATE* regulates Al tolerance in root tip cells by regulating citrate transport in roots and controls Al ion-activated citrate extrusion (Huang et al., 2009; Wu et al., 2014). The first Al-tolerance transcription factor identified in rice was *ART1*, a C2H2 zinc finger transcription factor that is localized in the root nucleus and is constitutively expressed in the roots. This gene isn’t affected by Al treatment and is homologous to *STOP1* in Arabidopsis (Yamaji et al., 2009; Tsutsui et al., 2011). Among the 31 genes regulated by the ART1 transcription factor, *STAR1* is the most important gene for Al tolerance in rice and, together, *STAR1* and *STAR2* encode a bacterial ABC transporter that transports UDP glucose to repair cell walls and thus enhances Al tolerance in rice.

Although some genes associated with Al toxicity in rice have been cloned and the mechanism of rice tolerance to Al toxicity has been speculated to involve components of the cell wall, research on the Al tolerance and detoxification mechanism of rice remains far from sufficient. In recent years, biotechnology has been increasingly used not only to study the complex physiological responses of many organisms to stress but also to elucidate the mechanisms of stress tolerance. In particular, integrated analyses involving various omics, such as transcriptomics, metabolomics, and proteomics, can provide important biological information in a more comprehensive manner (Chen et al., 2019; Solhaug et al., 2019; Gong et al., 2020; Li et al., 2021). Therefore, we used the differentially expressed genes (DEGs) identified from the transcriptome and the differential metabolites (DMs) identified from the metabolome to characterize the oxidative stress response of roots in rice landraces differing in degree of Al tolerance under exposure to Al stress. This study reveals a novel mechanism through which rice roots respond to Al stress and lays a theoretical foundation for the subsequent breeding of plants with Al tolerance.

## Materials and methods

### Plant materials and growth conditions

We previously screened Ting’s rice collection (one of the earliest rice accession collections in China) under 100 μM AlCl_3_ (.5 mM CaCl_2_, pH 4.0) stress and selected the two materials representing the largest difference in relative root elongation (RRE), comprising the Al-tolerant landrace Shibanzhan (KR; mean RRE = .860) and the Al-sensitive landrace Hekedanuo (MR; mean RRE = .299) (Zhang J et al., 2016; Zhao et al., 2018). In this study, KR and MR were subjected to transcriptomic and metabolomic analyses for Al tolerance.

We selected large, full seeds of KR and MR (400 seeds per landrace). The seeds were surface-sterilized by soaking in 5% H_2_O_2_ and shaken on a rotary shaker for 15–20 min at 150 rpm. After washing with distilled water 3–5 times to remove residual H_2_O_2_, the seeds were soaked in ddH_2_O at 26 ± 2°C in the dark for 24 h. The seeds for each landrace were divided into four groups of 100 grains, comprising one control group and three treatment groups. The treatment groups were pretreated with .5 mM CaCl_2_ (pH 4.0) at 30°C in the dark, and the solution was discarded after 24 h. Previous studies have shown that when seeds can provide all essential mineral nutrients, a simple CaCl_2_ solution method can be used to screen the Al^3+^ tolerance of young seedlings and avoid Al precipitation in Yoshida’s solution (Famoso et al., 2010; Yokosho et al., 2011). Under the condition of a constant pH of 4.0 and .5 mM CaCl_2_ solution, the seeds were treated with 0, 50, 100, or 200 μM AlCl_3_ in the dark at 30°C for 48 h. In the control group, the culture conditions were the same as those in the treatment group except that no Al^3+^ was added. After 48 h, the bud length (length from the base to the bud tip) and root length (length of the longest root) were measured and photographed (Zhao et al., 2018). The germinated seeds were placed into a plastic PCR plate with a hole depth of 1.5 cm with sterilized tweezers to expose the bud tip on the surface of the plate and cultured in distilled water for 1–6 days. The seedlings were then placed into a plastic basin containing Yoshida’s rice nutrient solution (International Rice Research Institute) and cultured in an incubator until the 11th day after germination. During this period, the distilled water or nutrient solution was changed every second day. The incubation environment was as follows: 12 h at 28°C with illumination (light intensity 2,500 lux), then 12 h at 24°C in the dark. On 11 day, the treatment group was pretreated with 500 μM CaCl_2_ (pH 4.0) for 24 h. After pretreatment, the solution was discarded, and the seedlings were treated with 100 μM AlCl_3_ (.5 mM CaCl_2_) rice nutrient solution for 48 h under constant pH of 4.0. The seedlings were then divided into two groups: one group was frozen in liquid nitrogen for transcriptomic and metabolomic analyses; the second group was dried and the Al ion content was measured using an atomic absorption spectrometer (Perkin Elmer Analyst 200). Rice nutrient solution lacking Al^3+^ (0 μmol/L) was used as the control. Each treatment group and the control comprised three biological replicates.

### Transcriptome sequencing and data analysis

The samples of the Al-tolerant landrace KR and Al-sensitive landrace MR under Al treatment were designated KR-Al and MR-Al, and those without Al treatment were designated KR-CK and MR-CK as controls. Three biological replicates were collected for each treatment. Five sites were randomly selected on the root of the seedlings for sampling and were then mixed. The root samples were immediately placed in liquid nitrogen and stored at −80°C.

Total RNA was isolated using a genomic RNA kit (CAT9637, TaKaRa), in accordance with the manufacturer’s instructions, from the three biological replicate samples. RNA quantity and quality were assessed by 1% agarose gel electrophoresis and with a Nanodrop spectrophotometer. After construction of the RNA library, a Qubit2.0 Fluorometer was used for preliminary quantification, and the library was sequenced on an Illumina HiSeq platform. Four fluorescent-tagged dNTPs, DNA polymerase, and primers were added to the flow cell for amplification.

FastQC was used to analyze the raw reads for quality control. High-quality clean data were used for subsequent analysis. All 12 libraries (KR-CK, KR-Al, MR-CK, and MR-Al, and three replicates for each treatment) were consolidated into one cistern. We used Trinity software (v.2.11.0) to assemble the rice root transcriptome. All assembled unigenes were annotated using the following databases: NR (NCBI non-redundant protein sequences), GO (gene ontology), and KEGG (Kyoto Encyclopedia of Genes and Genomes) pathways. The fragments per kilobase of transcript per million mapped reads (FPKM) value was used as an indicator of transcription or gene expression levels. The DESeq2 R package was used for the identification of the DEGs. Gene ontology (GO) analysis of the DEGs was conducted using the GOseq R package (GO terms with corrected *p*-value < .05 were significantly enriched among the DEGs). For GO enrichment analysis, we used the topGO Bioconductor package and KEGG pathway database.

### Real-time quantitative polymerase chain reaction analysis and candidate gene sequence alignment

We verified the measurement level of gene expression by real-time quantitative polymerase chain reaction (qPCR). Under Al stress, five DEGs randomly selected and two Al-tolerant candidate genes were subjected to qPCR analysis to demonstrate the accuracy and reproducibility of the transcriptomics data in this study (Supplementary Table S1). Primers for qPCR were designed using Premier 5. The RNAiso Plus kit (Takara, Dalian, China) was used to extract total RNA, and reverse transcription was carried out using the Prime Script RT Master Mix reverse transcription kit (Takara, Dalian, China) to synthesize complementary DNA (cDNA). In qPCR, Actin is the internal reference gene, and the system is as follows: ×2 SYBR GreenPro Taq HS Premix 10 μl; cDNA 4 μl; Primer F(10 μM) .8 μl; Primer R(10 μM) .8 μl; ROX Reference Dye(20 μM) .4 μl; Rnase free water to 20 μl. Finally, the relative gene expression was calculated with 2^−ΔΔCT^.

DNA Quick Plant System (Tiangen Biotechnology, Beijing, China) was used to extract plant genomic DNA. The target sequence was amplified with specific primers. The amplified product was purified with EasyPure PCR purification kit (Tiangen Biotech, China) and quantified with NanoDrop 8,000 spectrophotometer (Thermo Fisher Science, Waltham, MA, United States). Sequence alignment was performed with the DNAMAN software using the genes in the Nipponbare genome as a reference.

### Widely targeted metabolomic analysis

Quantification of metabolites was performed using multiple response monitoring (MRM) by MetWare Biotechnology Ltd. (Wuhan, China) (Yang et al., 2020). Linear ion trap (LIT) and triple quadrupole (QQQ) scans were performed with an API6500Q trap liquid chromatography–tandem mass spectrometry (LC-MS/MS) system. The electrospray ionization operating parameters were as follows: ion source turbine spray; source temperature 500°C; ion spray voltage 5500 V; ion source gas I, gas II, and curtain gas set to 55, 60, and 25.0 psi, respectively; and collision-activated dissociation was higher.

Metabolites were determined by comparing the *m*/*z* values, retention time, and fragmentation patterns with the standard in a database compiled by MetWare Biotechnology Ltd. The filtering criteria for the significantly changed metabolites (SCM) were |log_2_(fold change)| ≥ 1 and variable importance in the projection ≥1. Principal component analysis (PCA) of the SCMs was performed with R software (www.r-project.org/) to assess the landrace-specific accumulation of metabolites.

### Statistical analysis

The data was analyzed through Excel 2019 and SPSS 22.0. And the data were expressed as the mean ± standard deviation (SD) of the three biological replicates. The difference between mean values was analyzed by Duncan’s multiple range tests, and *p* < .05 was regarded as significant. The same alphabet indicates no significant difference among the treatment groups, different alphabet indicates significant difference.

## Results

### Al tolerance and associated physiological traits estimated for KR and MR

**FIGURE 1:**
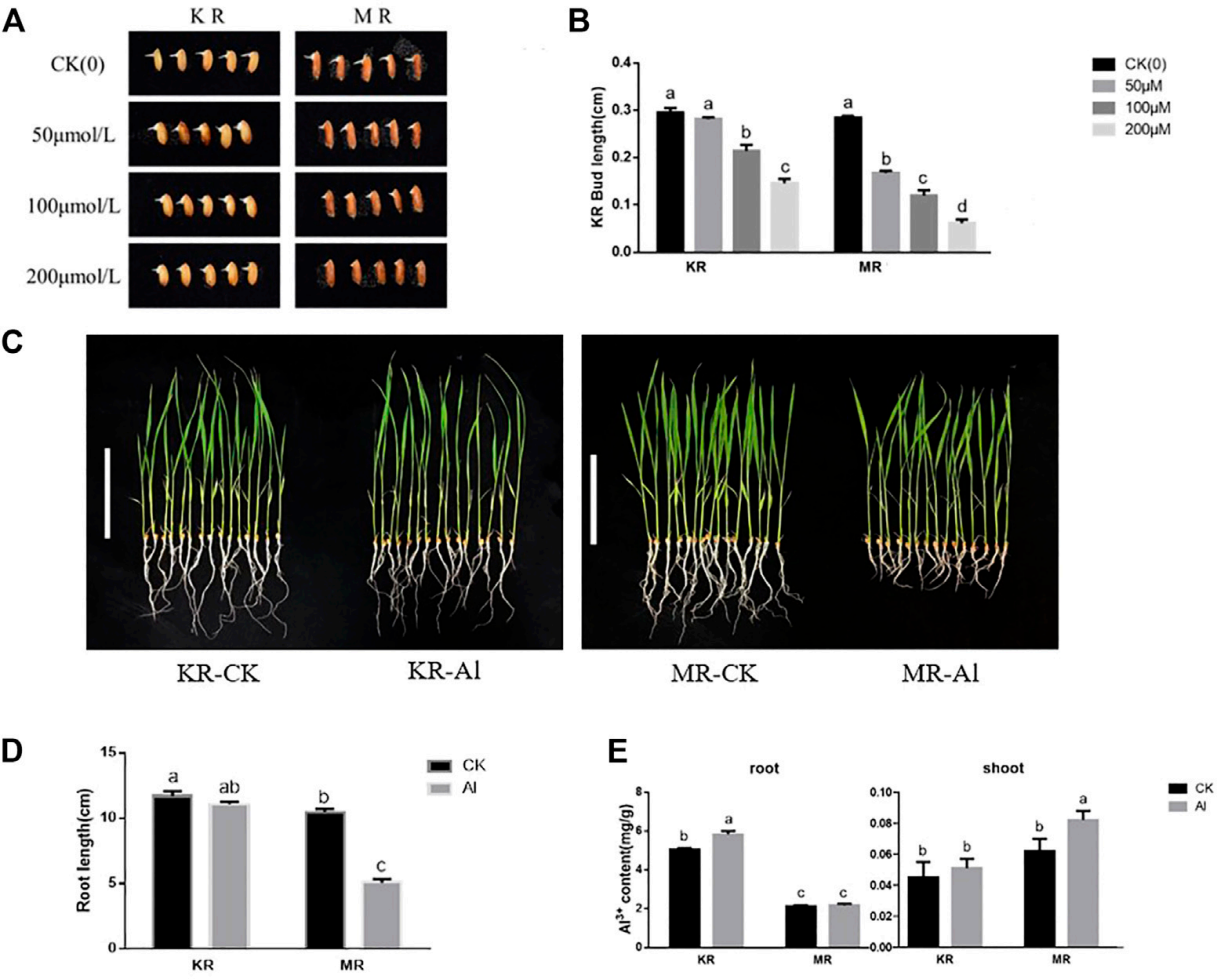
Bud length and root growth on rice landraces (KR-Al tolerant; MR-Al sensitive) under Al stress. **(A)** Effect by Al stress on Phenotype of rice at bud stage. **(B)** Bud length of rice under Al stress. **(C)** Effect by Al stress on Phenotype of rice seedlings (Bar represent the 10 cm). **(D)** Effect by Al stress on root length of rice seedlings. **(E)** The content of Al ions absorbed by rice seedling under Al stress.

To evaluate the degree of tolerance of KR and MR to Al stress, we identified the Al tolerance of 11 day-old seedlings at the budding stage. Under stress from 100 to 200 μM Al treatment, the bud lengths of KR and MR were significantly shorter than that of the control, and the bud length of KR was significantly longer than that of MR (Figures 1A,B). Compared with that of the control, the root length of KR decreased slightly under Al stress, whereas the root length of MR was significantly decreased (Figures 1C,D). Interestingly, the KR roots absorbed the highest amount of Al ions, and a significant difference in the amount of Al ions was observed between KR and the control. However, the amount of Al ions absorbed by MR roots increased only slightly and didn’t differ significantly from that of the control roots (Figure 1E). In contrast, only the content of Al ions in the aboveground parts of MR increased significantly under Al stress. These results suggested that the Al tolerance differed significantly between the two landraces.

### Identification of DEGs responsive to Al stress in KR and MR roots

**FIGURE 2:**
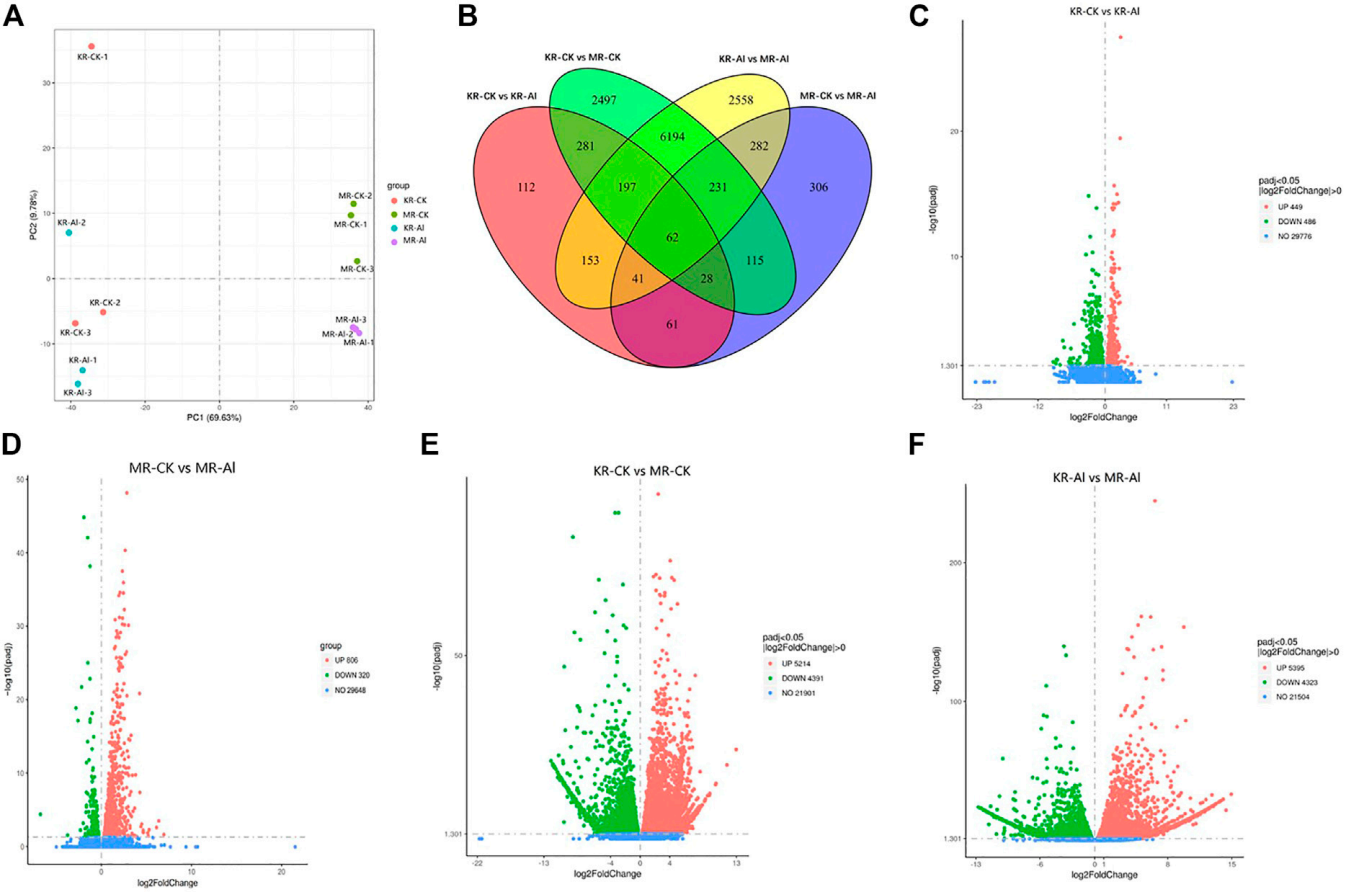
Transcriptomics changes of two rice landraces under Al stress. **(A)** PCA analysis of different treatment groups. **(B)** Venn graph for the comparisons of KR-CK vs. KR-Al and MR-CK vs. MR-Al. **(C)** Volcano plots of DEGs between KR-Al and KR-CK. **(D)** Volcano plots of DEGs between MR-Al and MR-CK. **(E)** Volcano plots of DEGs between KR-CK and MR-CK. **(F)** Volcano plots of DEGs between KR-Al and MR-Al. The dots: differential DEGs, Green dots: downregulated DEGs, Red dots: upregulated DEGs, Blue dots: detected DEGs without difference.

To investigate the molecular mechanism responsible for the difference in Al tolerance between KR and MR, we conducted transcriptomic analysis of the roots of KR and MR under Al stress. In total, 572 million clean reads with a length of 150 bp were generated. The clean reads ranged from 65.83% to 82.14%, and the average Q20 and Q30 values were 98.19% and 94.99%, respectively (Supplementary Table S2). The data for the same landrace was highly consistent (*R* ^2^ = .893–.982), indicating that the RNA-sequencing data were accurate and repeatable (Supplementary Figure S1). The PCA of the gene expression profiles of samples from the four treatments showed the distinction between the two landraces was largely accounted for by PC1 (which explained 69.63% of the total variance), whereas differences between the treatment groups and control group were largely accounted for by PC2 (which explained 9.78% of the total variance) (Figure 2A). A Venn diagram identified 935 DEGs (449 upregulated and 486 downregulated) in KR and 1126 DEGs (320 upregulated and 806 downregulated) in MR under Al stress (Figures 2B–F). The other two comparisons (KR-CK vs. MR-CK and KR-Al vs. MR-Al) identified 9605 (5214 upregulated and 4391 downregulated) and 9718 (5395 upregulated and 4323 downregulated) DEGs, respectively (Figures 2C–F). A heatmap visualized the differences in expression of DEGs in response to Al stress among the four groups (Supplementary Figure S2).

Some genes regulated by ART1 have been shown to be involved in the internal and external detoxification of Al at different cellular levels. Therefore, the expression differences in the ART1 homolog between the two landraces under Al stress was analyzed. The expression of *STAR1* was significantly upregulated by approximately two-fold under Al stress in both KR and MR, whereas the expression of *STAR2* wasn’t significantly different in the two landraces. This finding suggested that other Al-tolerance regulatory mechanisms may operate in the two rice landraces. In addition to *STAR1*, the expression of *Os10g0524600* (which encodes the subtilisin-like protease SBT1.2), *Os10g0206800* (which encodes the protein DETOXIFICATION 42), and four other genes was significantly upregulated in KR under Al stress. The expression of *Os12g0227400* (which encodes a 2-alkenal reductase (NADP(+)-dependent), *Os02g0186800* (which encodes a premnaspirodiene oxygenase), and 10 other genes was significantly upregulated in MR under Al stress. We noted that the expression of *Os02g0770800*, which encodes the nitrate reductase N*AD(P)H, w*as significantly downregulated by half in MR under Al stress (Supplementary Table S3). To verify the accuracy and repeatability of the transcriptomic analysis, five DEGs were randomly selected for quantitative real-time PCR (qPCR). The expression trends of the five DEGs were consistent with the transcriptomic data (Supplementary Figure S3), which indicated that the expression data in transcriptomic analysis were reliable.

### GO and KEGG enrichment revealed different molecular mechanisms of root response to Al stress in KR and MR

**FIGURE 3:**
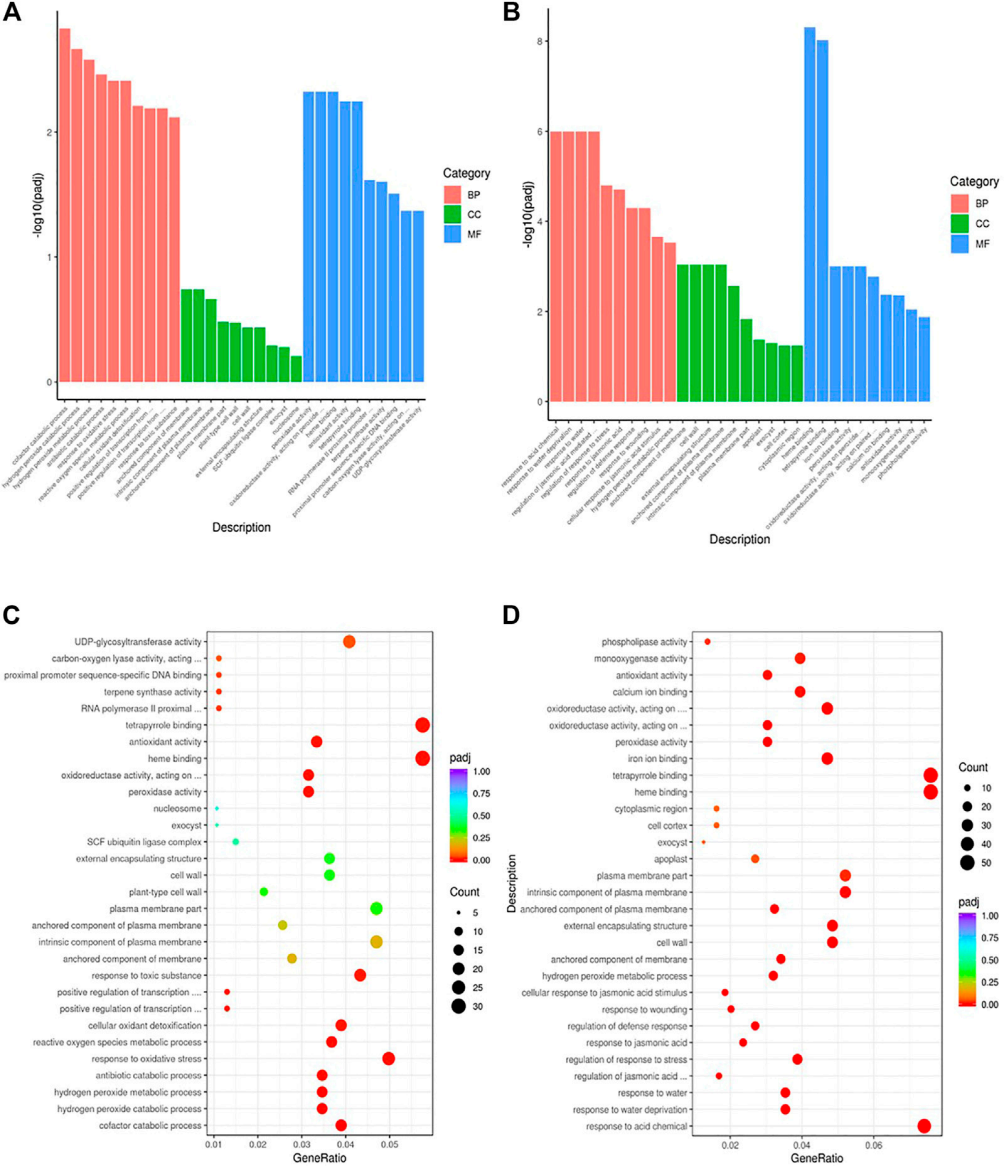
GO enrichment analysis of DEGs in roots of two rice landraces. **(A)** GO analysis of KR. **(B)** GO analysis of MR. **(C)** GO enriched analysis of KR. **(D)** GO enriched analysis of MR.

**FIGURE 4:**
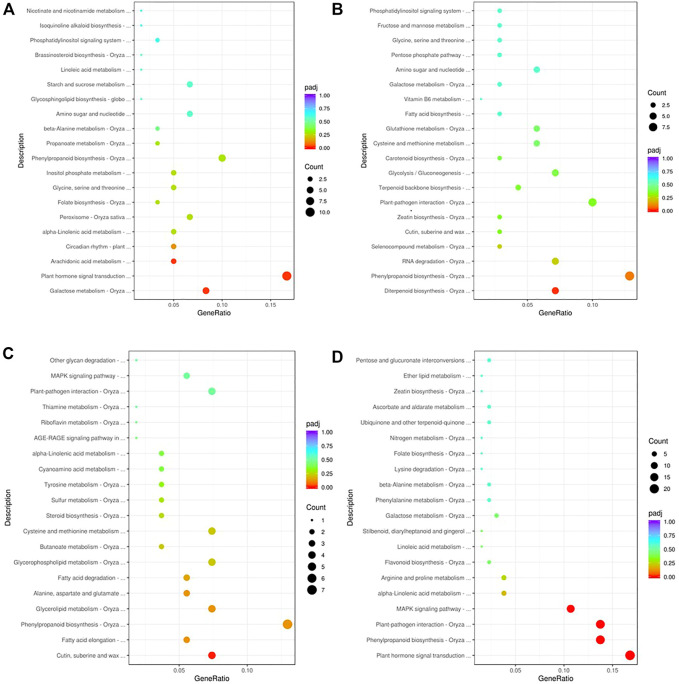
KEGG enrichment analysis of DEGs in roots of two rice landraces. **(A)** KEGG analysis of upregulated genes of KR. **(B)** KEGG analysis of downregulated genes of KR. **(C)** KEGG analysis of up-regulated genes of MR. **(D)** KEGG analysis of downregulated genes of MR.

To gain insights into the biological significance of the DEGs, we performed GO and KEGG enrichment analyses. Almost all DEGs were categorized into three categories: biological processes, cellular components, and molecular functions (Figures 3A,B). “Cofactor catabolic process” was the most highly enriched GO item in KR compared with the control group, and “response to acid chemical” was the most highly enriched GO item in MR compared with the control group (Figures 3C,D). Under Al stress, “galactose metabolism,” “plant hormone signal transduction,” and “arachidonic acid metabolism” were the most highly enriched KEGG pathways among the 449 upregulated DEGs in KR, whereas “cutin, suberine and wax biosynthesis” was the most highly enriched KEGG pathway for the 320 upregulated DEGs in MR. The KEGG pathways “diterpenoid biosynthesis” and “phenylpropanoid biosynthesis” were the most highly enriched for the 486 downregulated DEGs in KR, and “plant hormone signal transduction”, “phenylpropanoid biosynthesis”, “plant–pathogen interaction”, and “MAPK signaling pathway” were the most highly enriched for the 806 downregulated DEGs in MR compared with the control group (Figures 4A–D). The difference in phenylpropanoid biosynthesis was prominent in both KR and MR under Al stress, which indicated that phenylpropanoid biosynthesis might be an important pathway in the regulation of Al tolerance between the two landraces. Comparison of the phenylpropanoid biosynthesis pathway under Al stress showed that the expression of *Os06g0522300* (which encodes peroxidase 2-like), *Os06g0521900* (which encodes a lignin-forming anionic peroxidase), and *Os03g0234500* (which encodes peroxidase A2) was significantly upregulated in KR and MR.

### Metabolite analysis in KR and MR under Al stress

To understand the differences in Al tolerance between KR and MR at the metabolic level, we identified the alterations in metabolites in KR and MR roots by LC-MS/MS. The R2Y scores for the KR-Al vs. KR-CK and MR-Al vs. MR-CK comparisons were higher than 99 (Supplementary Figures S5B,C), indicating the accuracy of the experimental results. A heatmap showed the distinct hierarchical clustering of 550 metabolites (Supplementary Table S6; Supplementary Figure S5A) in the two landraces. OPLS-DA indicated that there were significant intragroup differences between the two landraces, and the contents of 88 metabolites in KR and 27 metabolites in MR differed significantly under Al stress (Supplementary Figure S4). Organic acids are considered to be the most important organic compounds in the tolerance of plant roots to Al stress. Investigation of the organic acids among the 115 differential metabolites (DMs) of KR and MR under Al stress revealed that the contents of 2, 3-dihydroxybenzoic acid and phenylacetate showed significant differences in KR, and their accumulation was double that in the control group. The contents of citraconic acid and fumaric acid showed significant differences in MR under Al stress, and their accumulation was reduced by half compared with that in the control group. These findings indicated that these four organic acids might be crucial metabolites involved in the differential tolerances of KR and MR to Al stress (Supplementary Tables S4, S5).

### Analysis of metabolic pathways of KR and MR roots response to Al stress

**FIGURE 5:**
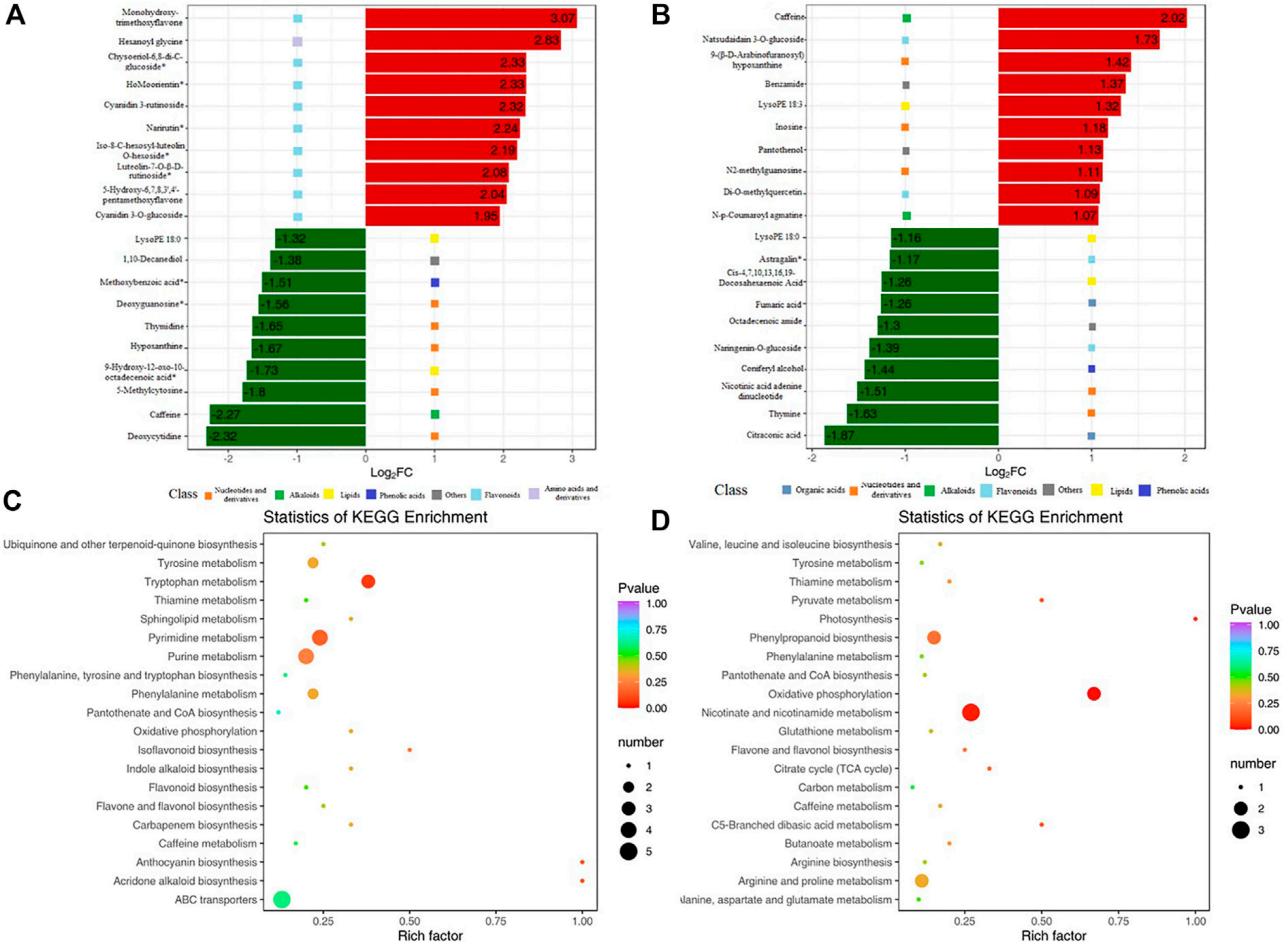
KEGG enrichment pathway of DMs and bar chart of top 10 DMs. **(A)**Top 10 upregulated and downregulated DMs for KR. Red bars means upregulated DMs. Green bars means downregulated DMs. **(B)**Top 10 upregulated and downregulated DMs for MR. **(C)** KEGG enrichment pathway of DMs for KR. **(D)** KEGG enrichment pathway of DMs for MR.

Compared with that in MR-Al, DMs in KR-Al were mainly enriched in the biosynthesis of plant secondary metabolites, such as nucleotides, flavonoids, and derivatives. The top 10 upregulated DMs in KR were mainly enriched in flavonoids, and the downregulated DMs were mainly enriched in nucleotides and derivatives under Al stress (Figure 5A; Supplementary Table S7). In MR, the top 10 up and downregulated DMs were mainly enriched in nucleotides and derivatives under Al stress (Figure 5B; Supplementary Table S7). Citraconic acid and fumaric acid were the top 10 downregulated DMs in MR under Al stress, and citraconic acid was the most strongly significant DM (Figure 5B; Supplementary Table S8).

KEGG enrichment pathway analysis revealed alterations in pathways of the biological processes category in the two landraces in response to Al stress. “Pyrimidine metabolism” and “tryptophan metabolism” showed significant differences in KR under Al stress, and “nicotinate and nicotinamide metabolism”, “oxidative phosphorylation”, and “phenylpropanoid biosynthesis” showed significant differences in MR (Figures 5C,D).

### Integrated transcriptomic and metabolomic analysis of KR and MR roots in response to Al stress

**FIGURE 6:**
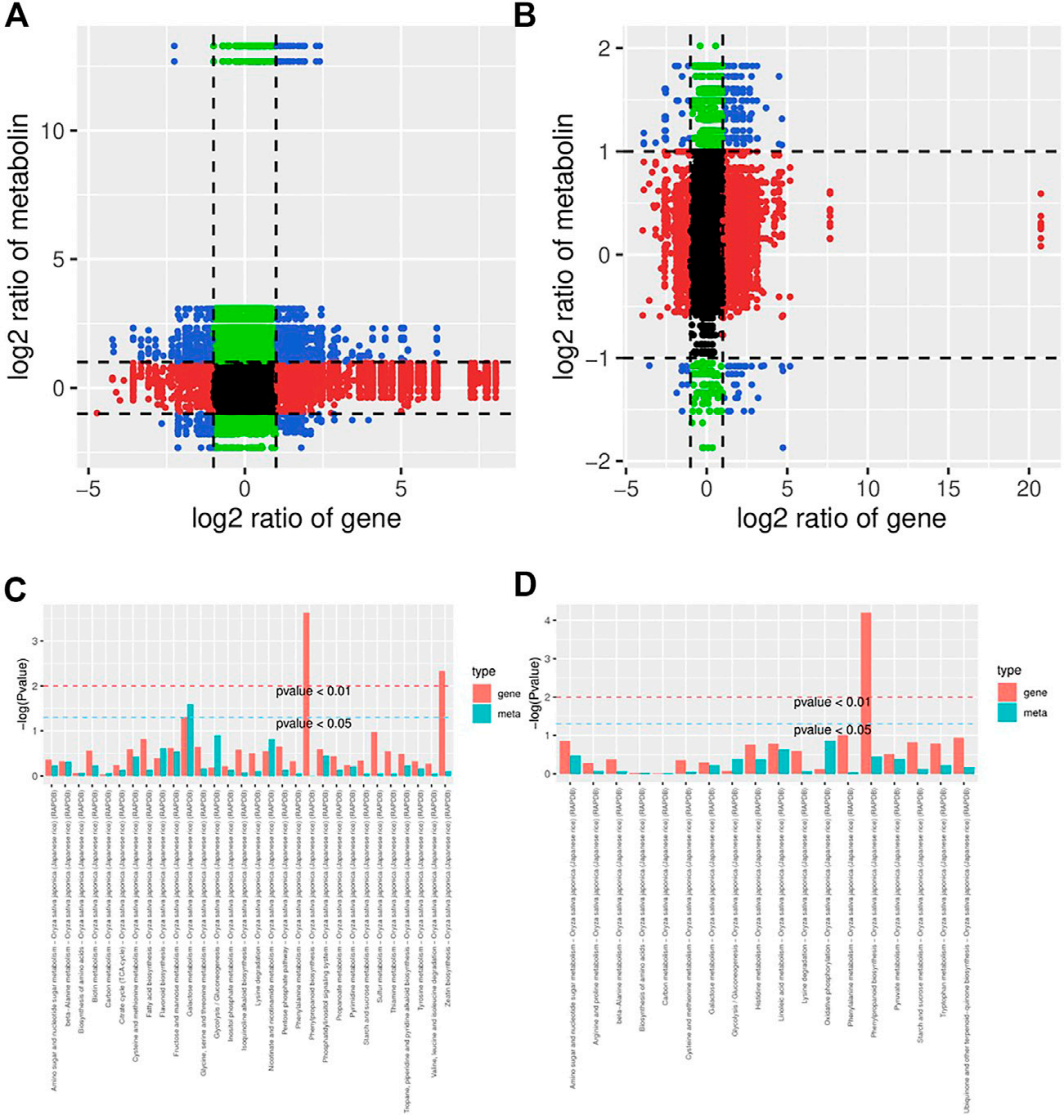
Network analysis of DEGs and DMs of rice roots following Al stress. **(A)** Nine-quadrant map of genes and metabolites of KR-CK vs. KR-Al. **(B)** Nine-quadrant map of genes and metabolites of MR-CK vs. MR-Al. **(C)** KEGG enrichment analysis of differential genes and metabolites in KR-CK vs. KR-Al. **(D)** KEGG enrichment analysis of differential genes and metabolites in MR-CK vs. MR-Al.

To further understand the response of KR and MR to Al stress, we analyzed the transcriptome and metabolome by constructing a coexpression network. A nine-quadrant diagram was generated to reveal the relationships among genes and metabolites (Figure 6A). The genes in the third and seventh quadrants exhibited the same differential expression as the metabolites, which indicated that the alteration in the accumulation of metabolites may be positively regulated by genes (Figures 6A,B). A heatmap, which had a Pearson correlation coefficient >.8, revealed the clustering characteristics of the DEGs and DMs (Supplementary Figure S6). The results showed that the DEGs accumulated in the “phenylpropanoid biosynthesis” pathway exhibited extremely significant differences in both KR and MR under Al stress (*p* < .01), which indicated that phenylpropanoid biosynthesis may play a role in tolerance to Al stress (Figures 6C,D).

### Effects of Al stress on phenylpropanoid biosynthesis in KR and MR roots

**FIGURE 7:**
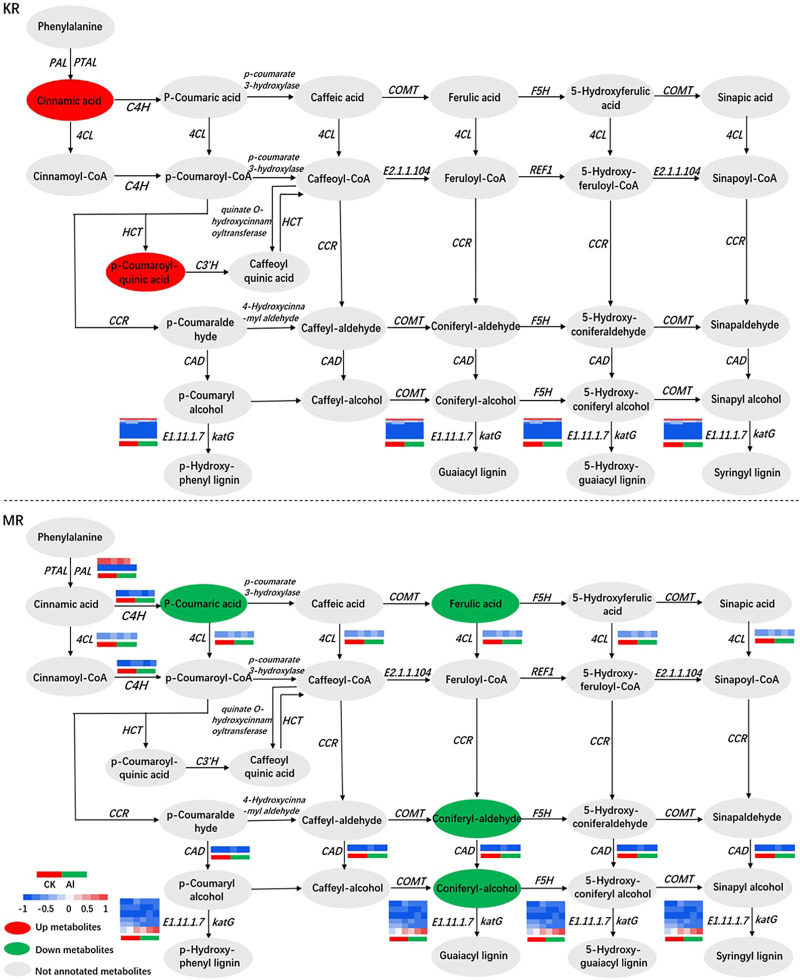
Correlation network of DEGs and DMs involved in phenylpropanoid biosynthesis.

Together, the results from the transcriptomic and metabolomic analyses revealed that phenylpropanoid biosynthesis may be the most sensitive pathway under Al stress. To understand the mechanisms of Al tolerance in KR and MR at the transcriptomic and metabolomic levels, we mapped the expression levels of genes and metabolites associated with phenylpropanoid biosynthesis (Figure 7). The contents of PAL, 4CL, and trans-cinnamate 4-monooxygenase (C4H) were significantly increased, and that of cinnamyl-alcohol dehydrogenase (CAD) was significantly decreased in MR under Al stress. Among the genes regulating E1.11.1.7 (peroxidase) in KR under Al stress, eight genes, including *Os06g0522300* and *Os03g0234500*, were upregulated. Three genes, including *Os04g0688100*, which encodes the cationic peroxidase SPC4, and *Os11g0112200*, which encodes the cationic peroxidase 1, were downregulated. In MR, five genes, including *Os06g0521900*, and *Os06g0521400*, which encodes peroxidase *P7*, were upregulated. *Os07g0531400*, which encodes peroxidase 39, was downregulated under Al stress (Figure 8; Supplementary Table S8). *Os06g0521900*, *Os06g0522300*, and *Os03g0234500* were upregulated in both KR and MR under Al stress. Among these genes, the expression of *Os03g0234500* under Al treatment was doubled compared with that in the control group and the expression under Al treatment was three times that in the control group for both KR and MR. Compared with that in the control, the expression of *Os06g0521900* in KR was increased by five times, and in MR was increased by two times, under Al stress (Supplementary Table S8). These results suggested that *Os06g0521900*, *Os06g0522300*, and *Os03g0234500* may be involved in the regulation of Al tolerance in rice. Although the expression of *Os06g0521900* was significantly upregulated in both KR and MR, the difference in KR was much higher than that in MR. Under Al stress, the contents of p-coumaroyl quinic acid and cinnamic acid were increased in KR, and the contents of p-coumaric acid, ferulic acid, coniferyl-aldehyde, and coniferyl-alcohol were decreased in MR.

### qPCR Validation of the candidate genes and sequence analysis

To verify whether the candidate genes were involved in the response to Al stress, qPCR was conducted on *Os06g0521900* and *Os02g0770800* (Supplementary Figure S7). The expression of *Os06g0522300* was upregulated in both KR and MR under Al stress, and the variation in KR was much higher than that in MR. The expression of *Os02g0770800* was downregulated in MR but not in KR under Al stress. Sequencing of two candidate genes in the landraces revealed that *Os06g0521900* had a common mutation site in KR and MR, that caused a change from glutamic acid to aspartic acid. In addition, four mutation sites were detected in MR, resulting in changes in three amino acids (Supplementary Figure S7C). *Os02g0770800* contained seven mutation sites in KR, causing three amino acid changes, whereas two insertion mutations were detected in MR, leading to premature termination of translation (Supplementary Figure S7D). These results supported the conclusion that *Os06g0521900* and *Os02g0770800* were involved in the response of rice to Al stress.

## Discussion

### Effects of Al stress on the genes and metabolites of two rice landraces

Previous studies have indicated that, under the pressure of limited nutrient availability, low temperature, and high salt, plants can regulate their tolerance to abiotic stresses through metabolic alteration and the regulation of DEGs (Ke et al., 2018; Tang et al., 2018). The Al tolerance of rice is regulated by a suite of genes that regulate external exclusion and internal detoxification mechanisms in response to Al stress and are regulated by ATR1 (Yamaji et al., 2009). ATR1 regulates at least 31 genes through core *cis*-acting elements in the promoter regions of these genes, and some of these 31 genes have been characterized (Tsutsui et al., 2011; Sun et al., 2021). *STAR1* and *STAR2*, which encode bacterial ABC transporters, act on the cell walls of plants and cause a decrease in Al accumulation in the cell wall, which is highly effective (Huang et al., 2009). In the present study, comparison of the expression of ATR1 homologs between the two landraces revealed that the expression of *STAR1* was doubled in both KR and MR, but expression of *STAR2* didn’t show significant differences under Al stress. The expression of *Os02g0770800* (which encodes a nitrate reductase) was decreased in MR but not in KR under Al stress, which was consistent with the results of Sharma and Dubey. (2005).

Organic acids (OAs) play many roles in plants, such as regulating stomatal closure (Vahisalu et al., 2008), nutrient absorption (Durrett et al., 2007), and defense against toxic ions (Pellet et al., 1995). Al-induced OA anion secretion from the roots of higher plants is a common mechanism of Al tolerance in plants. Distinct differences have been observed in the types, amounts, pathways, and sensitivities of OA anions secreted by different plants in response to Al stress (Ma, 2007). Ma et al. (2001) reported that malate, oxalate, and citrate play important roles in the Al tolerance of plants, and the chelating effect of citrate on Al ions was strongest in rice. Two 3-Dihydroxybenzoic acid can be dehydrogenated to salicylic acid, which plays a role in stress defense (Bartsch et al., 2010). Zhang P et al. (2016) observed that salt-tolerant wild soybean accumulate higher contents of citric acid under salt stress. The fumaric acid content of tolerant varieties increases significantly under abiotic stress (Matsunami et al., 2020). In the present study, 2, 3-dihydroxybenzoic acid and phenylacetate were significantly upregulated in KR under Al stress, whereas citraconic acid and fumaric acid were significantly downregulated in MR (Supplementary Tables S4, 5). These results suggest that KR roots produce more organic acids under Al stress to reduce the damage caused by Al toxicity, which also reveals why KR roots absorb more Al ions under Al stress and have stronger Al tolerance than those of MR.

### Effects of phenylpropanoid biosynthesis

In response to abiotic stress, most plants attain protect through phenylpropanoid metabolism, and the synthesized metabolites mainly include lignins and flavonoids (Gray et al., 2012; Kiani et al., 2024; Ruiz la Bastida et al., 2021). The main role of lignin is to accelerate the formation of xylem vessels by strengthening the cell wall and simultaneously transporting water and nutrients (Gray et al., 2012). The first three steps of phenylpropanoid biosynthesis are termed the general phenylpropanoid pathway (Dong and Lin, 2021). In this pathway, phenylalanine constitutes the starting point of the phenylalanine biosynthesis pathway involving PAL, phenylalanine/tyrosine ammonia-lyase (PTAL), C4H, and 4CL (Figure 7). The enzymes PAL and PTAL, which catalyze the first step in phenylalanine metabolism, convert phenylalanine into cinnamic acid. In addition, PAL is the critical enzyme in the phenylpropane metabolic pathway and lignin synthesis. The second step of the general phenylpropanoid pathway is the first oxidation reaction in the flavonoid synthesis pathway, which catalyzes the hydroxylation of cinnamic acid to p-coumaric acid (Wohl and Petersen, 2020). Third, 4CL catalyzes the formation of p-coumaroyl-CoA by adding CoA units to p-coumaric acid. CAD plays a role in the final stage of lignin monomer synthesis and is the crucial enzyme in lignin synthesis (Preisner et al., 2018). In the present study, KR and MR showed significant differences in the genes, but not metabolites, involved in phenylpropanoid biosynthesis after Al stress (Figures 6C, D). In MR, the contents of coniferyl alcohol and its aldehyde (which are precursors of the lignin polymer) and CAD were significantly reduced under Al stress, which may lead to the increased sensitivity of MR to Al stress. The expression of lignin-forming anionic peroxidase regulated by *Os06g0521900* is increased in response to abiotic stress (Ghosh Dasgupta et al., 2014). In the present study, the expression of *Os06g0521900* was significantly upregulated in both KR and MR under Al stress, and the difference in KR was more strongly significant (Supplementary Table S8), which was consistent with the findings of Ghosh Dasgupta et al. (2014). These results indicated that *Os06g0521900* may play a role in Al tolerance in rice.

## Conclusion

Molecular differences in resistance to Al stress between two rice landraces were investigated using a combination of transcriptomic and metabolomic analyses of the rice roots. Phenylpropanoid biosynthesis is considered to be the most significant pathway in response to Al stress. We speculated and preliminarily verified two genes that may be involved in the regulation of Al tolerance in rice: *Os02g0770800* and *Os06g0521900*. These results provide useful insights into the molecular mechanisms of Al detoxification in rice after Al stress. The focus of this study was to examine a series of metabolomic and transcriptomic changes in rice roots under Al stress. Therefore, it is necessary to further study the effect of Al poisoning on the crop yield and quality as well as the molecular mechanism of related genes regulating Al tolerance in rice under Al stress.

## Data Availability

The data presented in the study are deposited in the CNCB genome sequence archive (GSA) repository, accession number CRA008536.

## Supplementary material

SUPPLEMENTARY FIGURE S1 Correlation evaluation of biological repetition for transcriptomics analyses of two rice landraces under Al treatments.

SUPPLEMENTARY FIGURE S2 Heatmap of DEGs expression in KR-CK, MR-CK, KR-Al, MR-Al.

SUPPLEMENTARY FIGURE S3 Effect of Al stress on expression of 5 genes.

SUPPLEMENTARY FIGURE S4 OPLS-Score and OPLS-DA S-plot: **(A)** KR-CK vs KR-Al. **(B)** MR-CK vs MR-Al. **(C)** KR-CK vs KR-Al. **(D)** MR-CK vs MR-Al.

SUPPLEMENTARY FIGURE S5 Preliminary analysis of metabolomics data: **(A)** A total of 552 differential metabolites were detected. **(B)** OPLS-DA of KR-Al vs KR-CK. **(C)** OPLS-DA of MR-Al vs MR-CK.

SUPPLEMENTARY FIGURE S6 Heatmap of DEGs and DMs in the comparison of KR-Al vs. MR-Al.

SUPPLEMENTARY FIGURE S7 qPCR and sequencing verification of two Al resistant candidate genes in KR and MR roots. **(A)** qPCR in KR. **(B)** qPCR in MR. **(C)** Sequence analysis of *Os06g0521900*. **(D)** Sequence analysis of *Os02g0770800*.

SUPPLEMENTARY TABLE S1 qRT-PCR analysis for primers.

SUPPLEMENTARY TABLE S2 RNA-Seq statistics of KR and MR roots.

SUPPLEMENTARY TABLE S3 Expression changes of ART1-regulated genes in the root of KR and MR.

SUPPLEMENTARY TABLE S4 The relative content of metabolites in roots of KR.

SUPPLEMENTARY TABLE S5 The relative content of metabolites in roots of MR.

SUPPLEMENTARY TABLE S6 Widely targeted metabolomics analyses in KR and MR roots response to Al stress.

SUPPLEMENTARY TABLE S7 Top ten DMs in rice roots in the comparison of KR and MR.

SUPPLEMENTARY TABLE S8 Expression levels of DEGs involved in phenylpropanoid biosynthesis analyzed by transcriptomics.

## Abbreviations

Al, aluminum; KR, Shiban zhan; MR, Hekedanuo; DEGs, differentially expressed genes; DMs, differential metabolites; qPCR, quantitative real-time polymerase chain reaction; PAL, Phenylalanine ammonia-lyase; 4CL, 4-coumarate-coa ligase; ALTM, Malate transporter; PCA, principal component analysis; PLS-DA, partial least squares discriminant analysis; E2.4.1.82, raffinose synthase (EC:2.4.1.82); GOLS, inositol 3-alpha-galactosyl transferase; PFK, 6-phosphofructokinase; E1.11.1.7, peroxidase (EC:1.11.1.7); CAD, cinnamyl-alcohol dehydrogenase; C4H, trans-cinnamate 4-monooxygenase; NR, NCBI non-redundant protein sequences; GO, gene ontology; KEGG, Kyoto encyclopedia of genes and genomes; FPKM, fragments per kilobase of transcript per million mapped reads; LIT, Linear ion trap; QQQ, triple quadrupole; GSI, GAS I; GSII, Gas II; SD, standard deviation; OA, organic acids.
