# Supplementary material for: Transcriptomics and metabolomics reveal tolerance new mechanism of rice roots to Al stress
Source: Front Genet. 2023 Jan 10;13:1063984. doi: 10.3389/fgene.2022.1063984 (PMC9871393; doi:10.3389/fgene.2022.1063984)

Supplementary figure S1

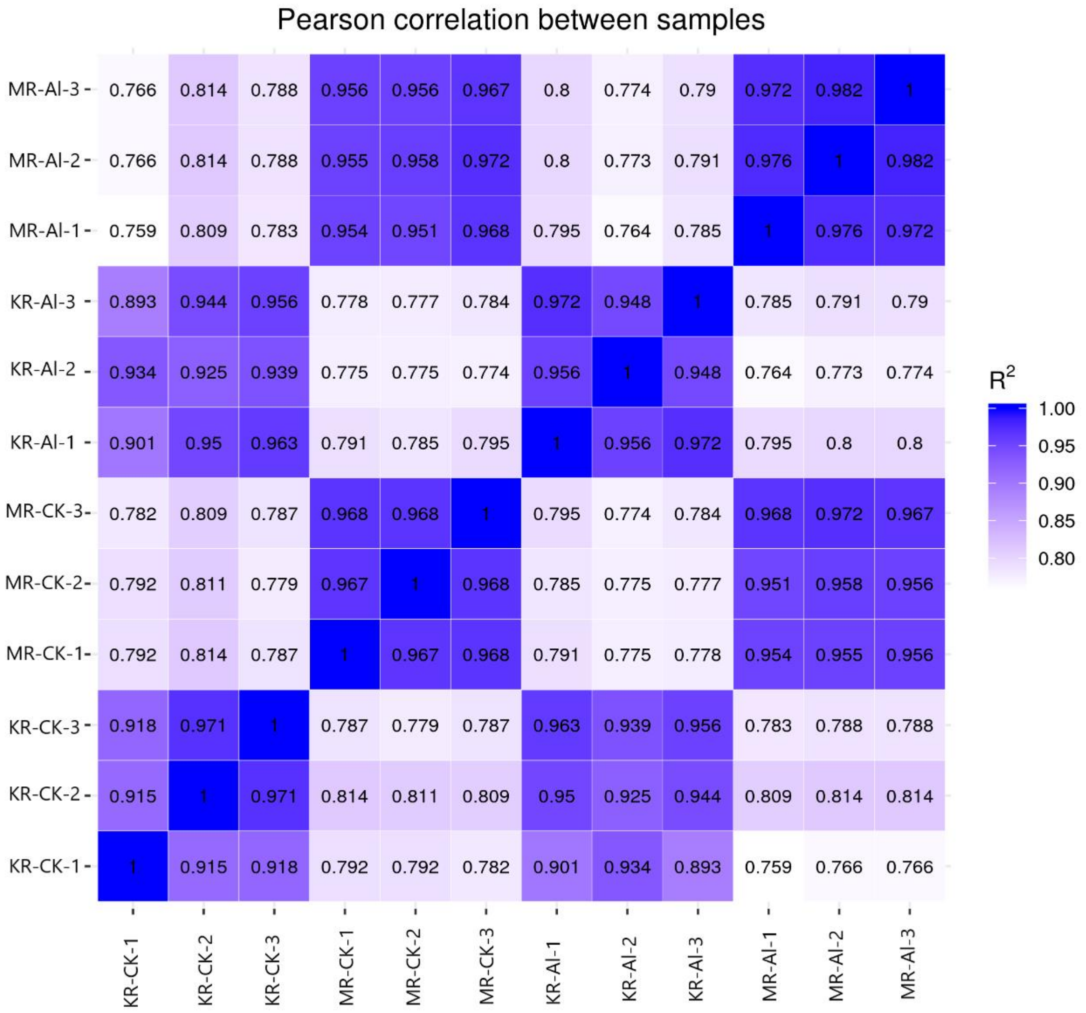

Supplementary figure S2

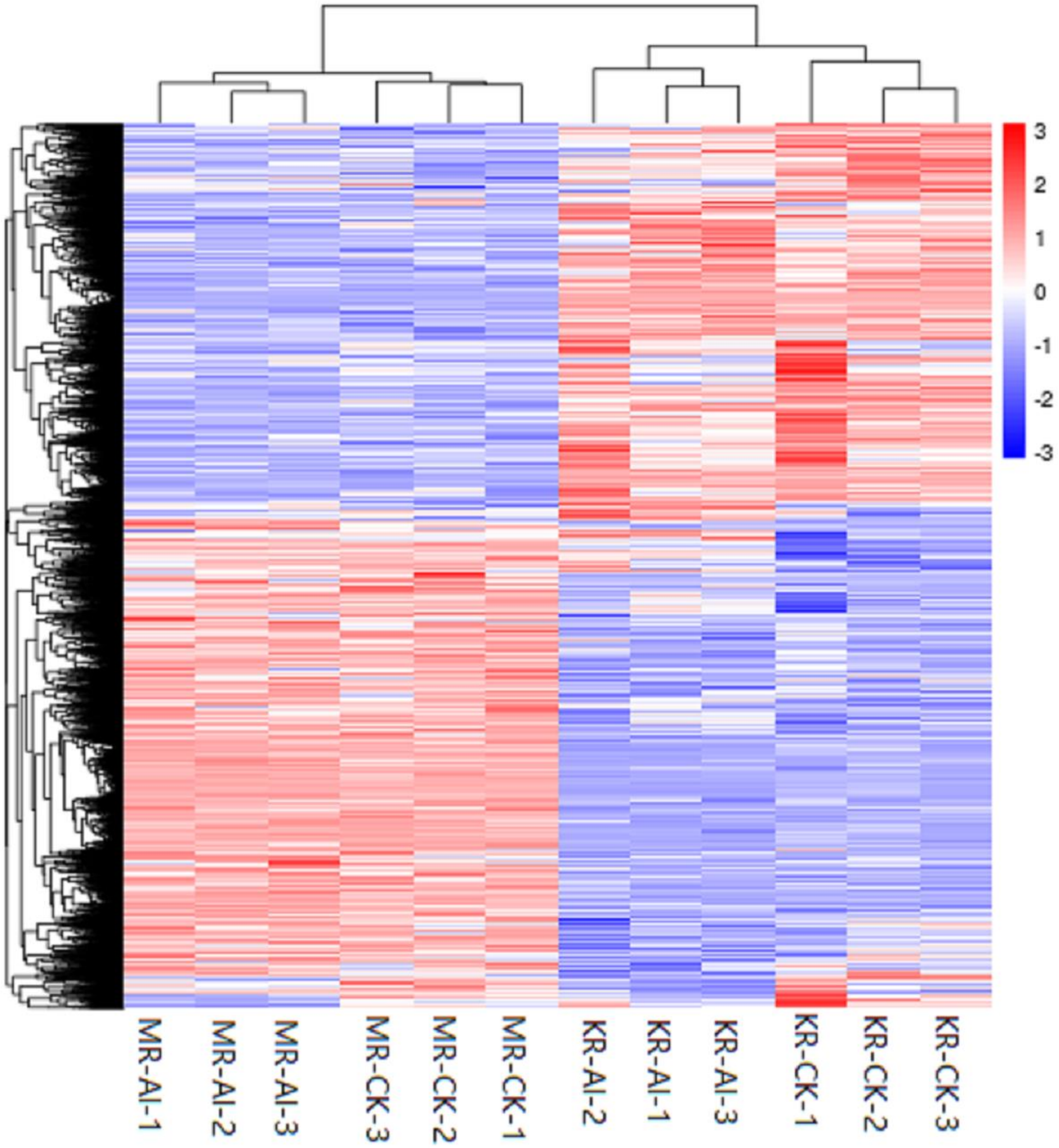

Supplementary figure S3

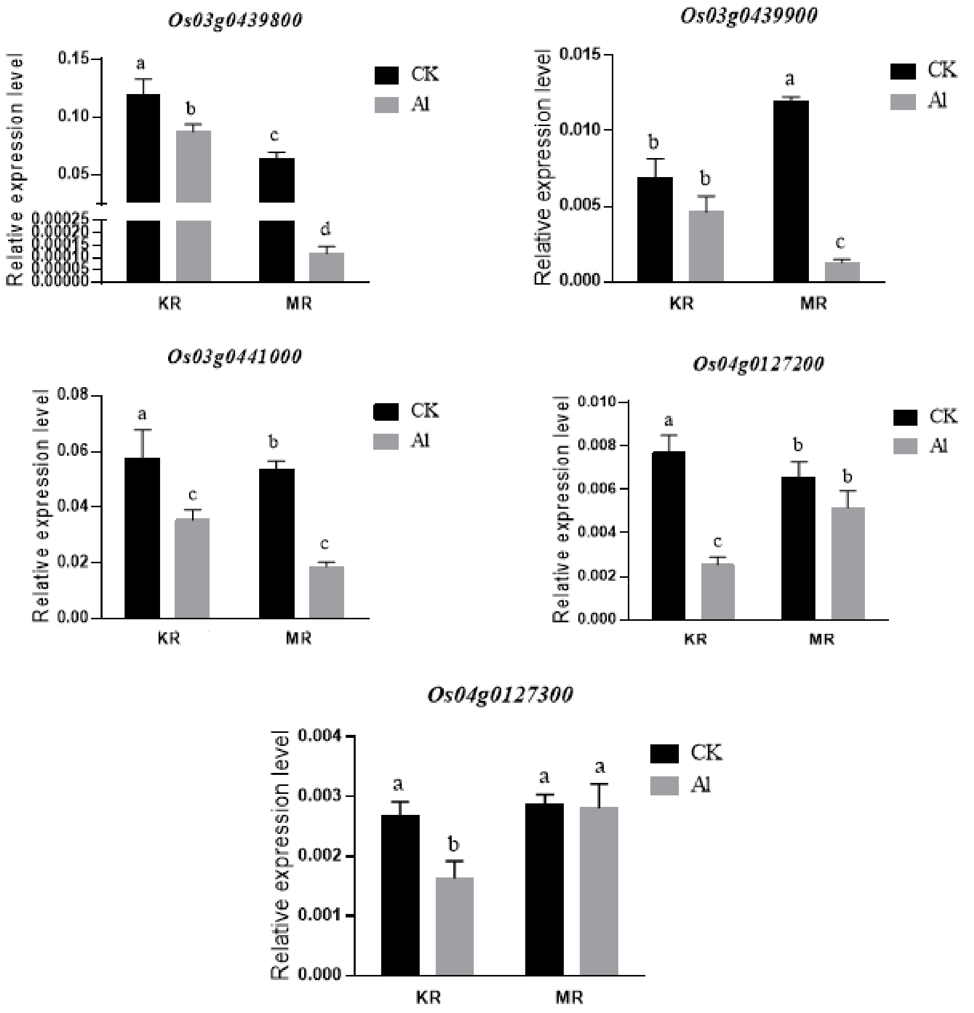

Supplementary figure S4

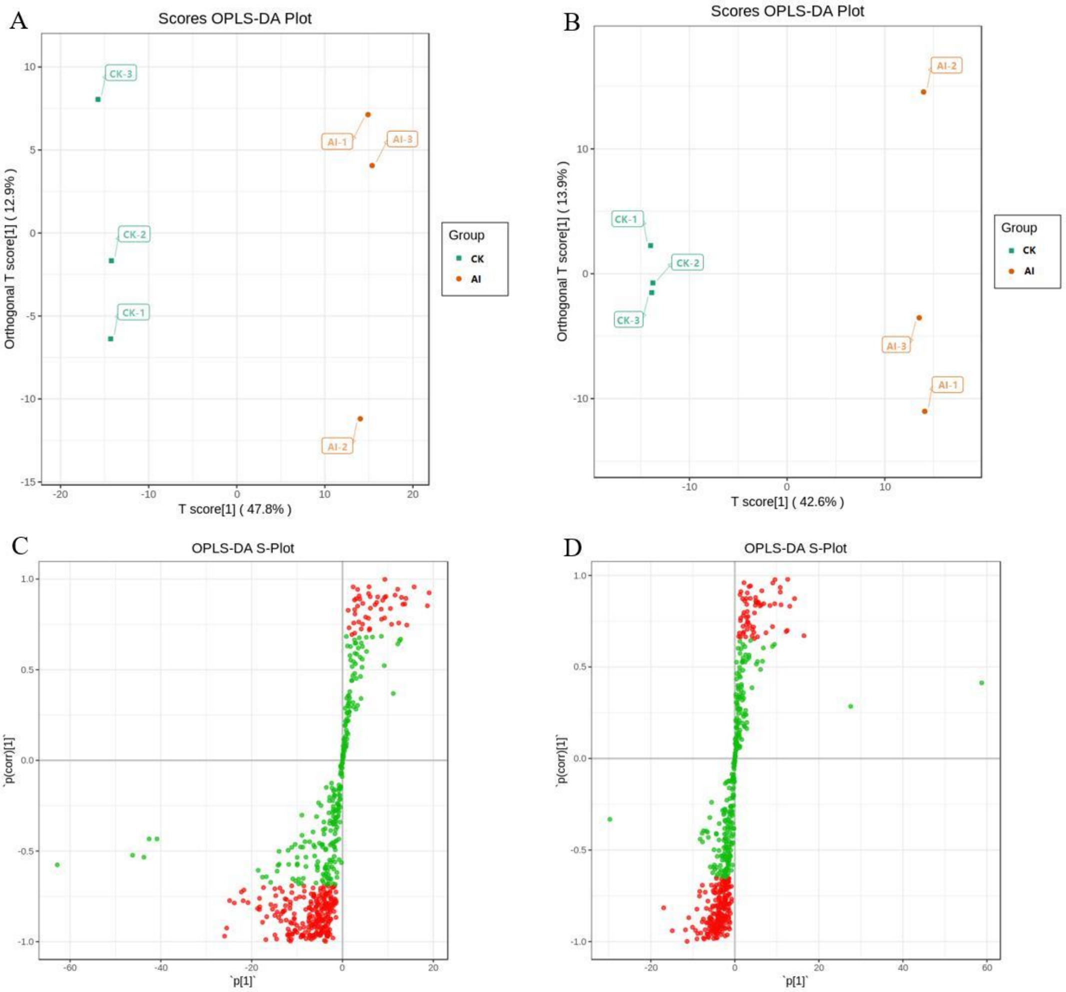

Supplementary figure S5

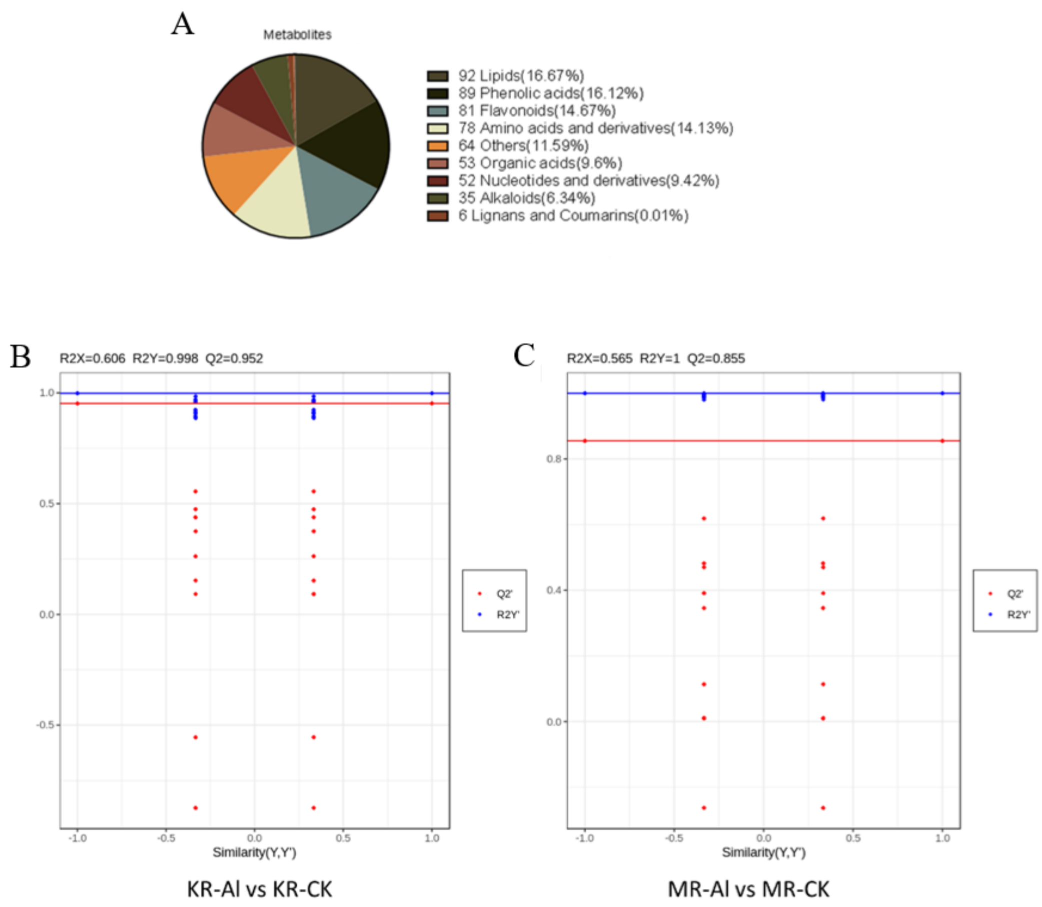

Supplementary figure S6

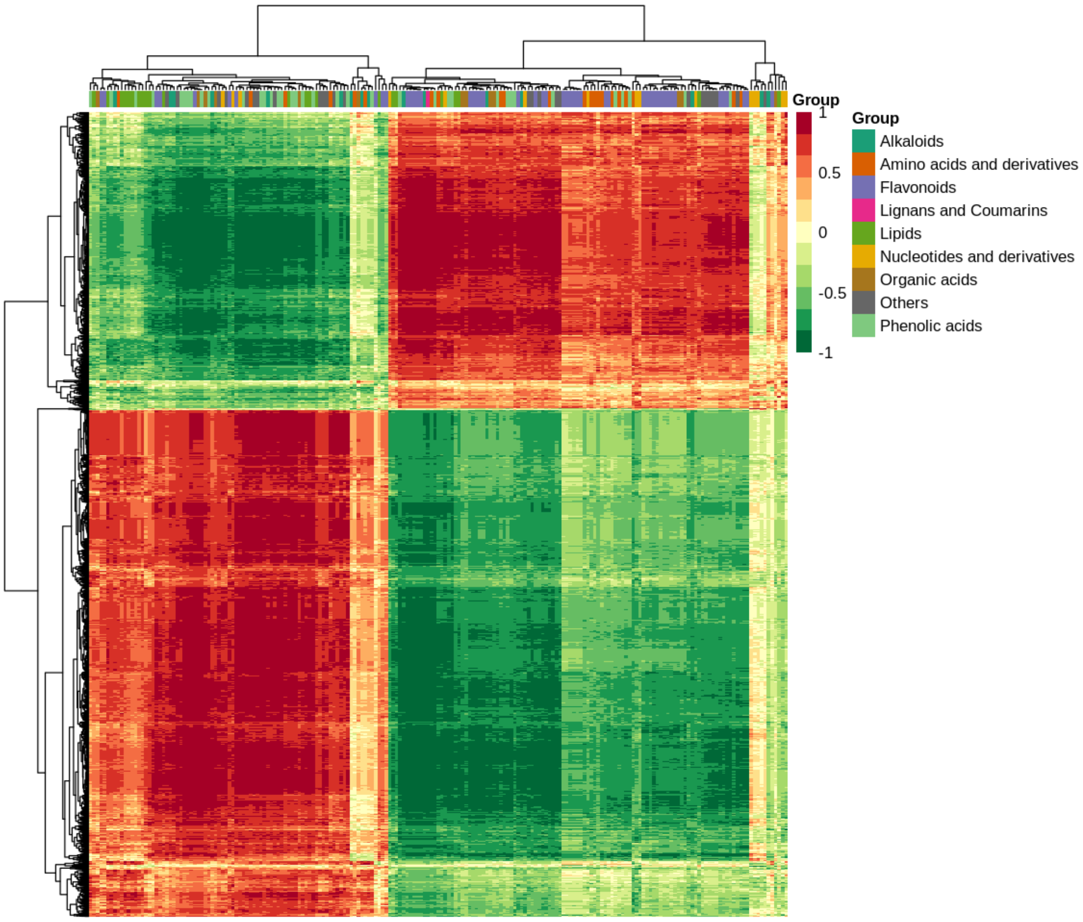

Supplementary figure S7

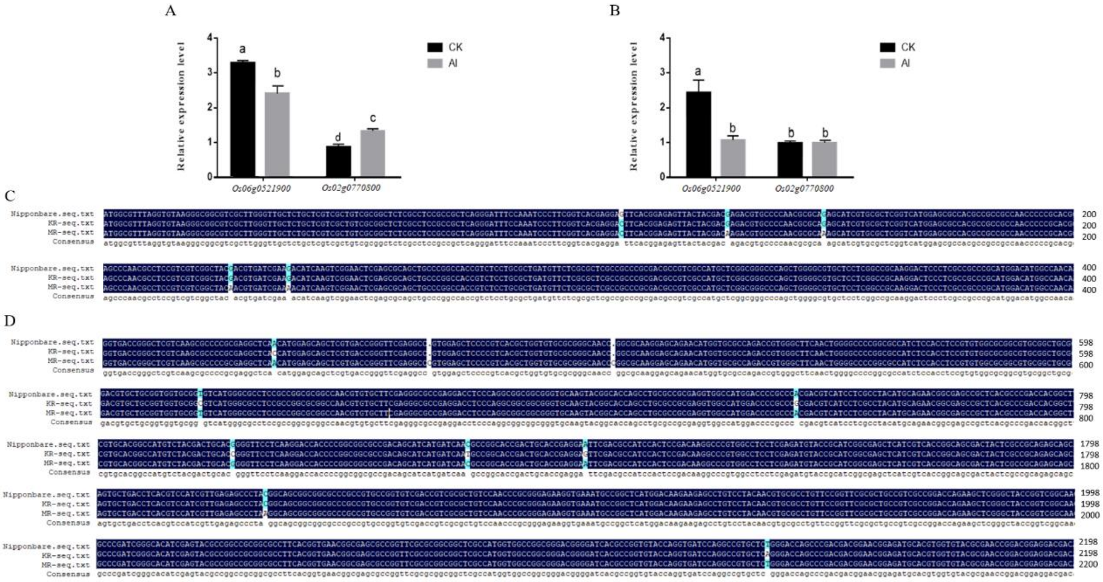

Supplement: Supplementary file 1 [file DataSheet1.PDF]
